# Evaluating surgical expertise with AI‐based automated instrument recognition for robotic distal gastrectomy

**DOI:** 10.1002/ags3.12784

**Published:** 2024-02-27

**Authors:** James S. Strong, Tasuku Furube, Masashi Takeuchi, Hirofumi Kawakubo, Yusuke Maeda, Satoru Matsuda, Kazumasa Fukuda, Rieko Nakamura, Yuko Kitagawa

**Affiliations:** ^1^ Department of Surgery Keio University School of Medicine Tokyo Japan; ^2^ Harvard College Harvard University Cambridge Massachusetts USA

**Keywords:** automated instrument recognition, gastric cancer, robotic distal gastrectomy, surgical skill

## Abstract

**Introduction:**

Complexities of robotic distal gastrectomy (RDG) give reason to assess physician's surgical skill. Varying levels in surgical skill affect patient outcomes. We aim to investigate how a novel artificial intelligence (AI) model can be used to evaluate surgical skill in RDG by recognizing surgical instruments.

**Methods:**

Fifty‐five consecutive robotic surgical videos of RDG for gastric cancer were analyzed. We used Deeplab, a multi‐stage temporal convolutional network, and it trained on 1234 manually annotated images. The model was then tested on 149 annotated images for accuracy. Deep learning metrics such as Intersection over Union (IoU) and accuracy were assessed, and the comparison between experienced and non‐experienced surgeons based on usage of instruments during infrapyloric lymph node dissection was performed.

**Results:**

We annotated 540 Cadiere forceps, 898 Fenestrated bipolars, 359 Suction tubes, 307 Maryland bipolars, 688 Harmonic scalpels, 400 Staplers, and 59 Large clips. The average IoU and accuracy were 0.82 ± 0.12 and 87.2 ± 11.9% respectively. Moreover, the percentage of each instrument's usage to overall infrapyloric lymphadenectomy duration predicted by AI were compared. The use of Stapler and Large clip were significantly shorter in the experienced group compared to the non‐experienced group.

**Conclusions:**

This study is the first to report that surgical skill can be successfully and accurately determined by an AI model for RDG. Our AI gives us a way to recognize and automatically generate instance segmentation of the surgical instruments present in this procedure. Use of this technology allows unbiased, more accessible RDG surgical skill.

## Graphical abstract

In the present study, we demonstrated that AI can be used to successfully identify and outline the surgical instruments used in robotic distal gastrectomy (RDG) with accuracy using novel instance segmentation. We further concluded that our AI model can accurately predict the surgical skill of a surgeon performing RDG by analyzing this surgical instrument usage.

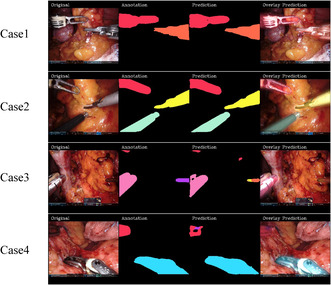

## 1 INTRODUCTION

Gastric cancer is one of the most common solid malignancies and the second leading cause of cancer deaths worldwide with nearly 800 000 deaths in 2020.^1^, ^2^, ^3^ Surgical resection with complete lymphadenectomy is the treatment that provides the best chance for survival and gastric cancer cure. Laparoscopic gastrectomy for gastric cancer was first introduced by Kitano et al. in 1994, and since then, minimally invasive approaches have been adopted rapidly in the treatment of gastric cancer including the introduction of the first robotic gastrectomy by Hashizume et al. in 2002.^4^, ^5^ The application of minimally invasive techniques has afforded patients many benefits including earlier recovery, fewer complications, and quicker initiation of adjuvant treatment when needed, with equivalent oncologic outcomes compared to open approaches as proven by multiple randomized prospective trials.^6^, ^7^, ^8^, ^9^, ^10^, ^11^, ^12^ As such, robotic distal gastrectomy (RDG) is becoming an increasingly widespread modality of treatment for resectable gastric cancer.^13^ Nevertheless, the surgical skill required for this procedure can vary greatly between practicing surgeons depending on factors such as hospital volume and prior surgical experience, and this can lead to differences in rates of postoperative morbidities and mortality.^14^, ^15^, ^16^ Variance in outcomes has been observed between certified experienced surgeons and non‐certified inexperienced surgeons, and thus surgical skill for distal gastrectomy has previously been quantified via laparoscopic video review.^17^, ^18^ For these reasons, assessing surgical skill in videos of RDG is critically important, both to assure quality and reproducibility along with being a valuable educational instrument for learning D2 lymphadenectomy.

In order to grade the surgical skill involved in performing RDG, previous large‐scale studies have used experienced surgeons to manually review the robotic videos of the assessed physician.^17^, ^18^, ^19^ This requires several independent reviewers to watch one operation, a process that is both timely and collaboratively intensive. Therefore, the use of artificial intelligence (AI) to assess surgical skill potentially faster and with more standardization would provide an advantage over the alternative of human video review.

Recently, the application of AI has been growing rapidly in the medical field. AI is readily being used in imaging and video analysis with the capability of providing real‐time clinical decision making in multiple procedures such as endoscopic tumor diagnosis, laparoscopic cholecystectomy anatomic recognition, and pulmonary lesion detection.^20^, ^21^, ^22^ We earlier demonstrated the usability for AI in surgical fields for both laparoscopic and robotic approaches.^23^, ^24^, ^25^, ^26^, ^27^ Based on our prior research experience, we hypothesized that this state‐of‐the‐art technology can be applied to the evaluation of surgical skill. So far, two different strategies have been implemented by institutions including our own aimed to assess surgical skill. The first approach measures surgical skill by analyzing the surgical field and surgical phase of previously recorded videos.^23^ The second approach utilizes surgical instrument recognition, the method we chose to employ.^28^, ^29^, ^30^, ^31^, ^32^ To our knowledge, this study is the first to use AI surgical tool recognition to determine surgical skill in RDG.

In this present study, we aim to assess surgical instrument usage via a novel approach using instance segmentation to analyze the surgical skill in RDG. Instance segmentations are unique because they generate frame‐specific outlines of instruments in each frame, and we hypothesized that this more precise detection of robotic and surgical instruments will more accurately reflect the surgical skill determined by our model.

## 2 METHODS

### 2.1 Data sets

In this retrospective study, we evaluated a consecutive cohort of 55 patients who underwent RDG with D1+ lymph node dissection (LND) in nine cases and D2 LND in 46 cases for gastric cancer at Keio University Hospital in Tokyo, Japan, between 2018 and 2021. The patient's clinical characteristics, including age, sex, clinical findings, and short‐term outcomes, were retrospectively extracted from the hospital's electronic records. This study was approved by the Ethics Committee of Keio University School of Medicine, and informed consent was obtained from all patients.

### 2.2 RDG procedure

The surgical indications and extent of LND were determined based on the Japanese Gastric Cancer Treatment Guidelines.^33^ The RDG procedures were performed with the patient in the supine position, utilizing the da Vinci Xi system (Intuitive Surgical, Sunnyvale, California, USA). The surgeries were conducted by four board‐certified surgeons, and the da Vinci Xi system required four ports with an additional port used for the assistant.

The RDG procedure involved the following steps which have been described in our previous report.^23^ After entering the abdominal cavity, the omentum was incised approximately 3 cm from the stomach wall, extending towards the spleen. The incision continued until the left gastroepiploic vessels, which were then divided. Omentum dissection was continued towards the right and down to the transverse colon. The right gastroepiploic vein was divided just above the bifurcation of the anterior superior pancreaticoduodenal vein and right gastroepiploic vein. Following the division of the right gastroepiploic artery, the pre‐pancreatic soft tissues were removed. Supraduodenal lymph node dissection was performed, and the duodenum was transected using a 60‐mm stapler. Suprapancreatic lymph node dissection involved the common hepatic lymph node dissection, celiac lymph node dissection, and left gastric lymph node dissection. In cases where D1+ LND was performed, proximal splenic and hepatoduodenal LND were omitted. The lymph node on the lesser curvature side of the gastric wall was completely removed before transecting the stomach using two or three 60‐mm staplers. The choice of reconstruction technique, either Billroth‐I or Roux‐en‐Y, was based on the surgeon's preference, with Roux‐en‐Y being preferred for cases with a small remnant stomach. We mainly used the Maryland bipolar forceps, medium‐large clip applier, da Vinci Harmonic ACE and SureForm 60 instrument on the 3rd arm, the Fenestrated bipolar forceps on the 1st arm, and the Cadiere forceps on the 4th arm. The 8 mm endoscope plus, 30° was used on the 2nd arm. Based on the surgeons' preference, suction tube, laparoscopic grasper, and laparoscopic stapler were used by the assistant surgeon through the assistant port located between the 1st and 2nd arm.

### 2.3 AI model for surgical skill evaluation using instrument recognition

To establish the AI model for surgical skill evaluation using instrument recognition, two sequential steps were performed as follows.

Step 1: The establishment of automatic instrument recognition using AI.
Step 2: The comparison between experienced and non‐experienced surgeons based on usage of instruments during infrapyloric LND.

#### 2.3.1 Step 1: The establishment of automatic instruments recognition using AI

Some instruments including the Maryland bipolar forceps, Medium‐large clip applier, da Vinci Harmonic ACE, Stapler, Fenestrated bipolar forceps, and Cadiere forceps were used during the operation by the surgeon while the Suction tube was operated by the assistant: all these instruments were annotated manually. A surgeon (MT) manually extracted 1383 images from 55 videos, 1234 for training and the remaining 149 for testing. Extraction was done randomly, but images containing at least one instrument were selected. JS and TF performed annotations manually and independently. Discrepancies in the annotation were addressed by discussion between them. Finally, MT who is a board‐certified surgeon confirmed all the annotations.

All modeling procedures were performed using a script written in Python 3.7. Further, a computer equipped with an NVIDIA Geforce RTX 3090 graphics‐processing unit (NVIDIA; Santa Clara CA) and an Intel(R) Core (TM) central processing unit i9‐10 900X @ 3.70 GHz with 128‐GB random access memory (RAM) were utilized for model training. DeepLab v3 plus was utilized for the semantic segmentation task. Pretraining was performed on the ImageNet 2012 classification database, which contains 1.28 million images of general objects such as animals, scenes (e.g., beach, mountain), and food. Data augmentation was performed using Random LR flip and crop.

In order to assess the model, we used the two metrics: intersection over union (IoU) and accuracy. The IoU is a metric used to evaluate how well a deep learning network's predicted segmentation mask aligns with the ground truth annotated data. Also known as the Jaccard index, IoU is calculated by dividing the overlap between the predicted segmentation mask and the ground truth with the union of these two sets. The IoU ranges from zero to one inclusive [0, 1], where an IoU of one indicates the predicted area and ground truth are identical, and an IoU of zero indicates no overlap between the predicted and ground truth segmentation map. Accuracy, on the other hand, is a metric used to describe how well a model can classify different objects. It is calculated by finding the ratio between the number of correct predicted instances compared to the number of total predictions. These two metrics together can both provide information about how well the computer can draw boundaries around a surgical instrument (IoU) and if it classifies it as the correct surgical instrument (accuracy).

#### 2.3.2 Step 2: The comparison between experienced and non‐experienced surgeons based on usage of instruments predicted by AI during infrapyloric LND

To investigate the relationship between surgical skill and usage of instruments, we focused on identifying the number of appearances for each instrument during the procedure. We defined this value as the number of times a specific instrument is more than one pixel during the procedure, and this was predicted by the AI model. In order to calculate this, the AI made a prediction at a rate of one frame per second, resulting in the number of appearances throughout the duration of the video. To evaluate surgical skill precisely, images were extracted and predicted by AI only during the infrapyloric LND stage of the procedure, the most demanding step when performing RDG. The infrapyloric LND step is defined as being the beginning omental incision in the direction of the right gastroepiploic vein to the end of the duodenal resection.

Each of the four surgeons' surgeries were divided into two groups based on the order of their level of expertise: the “non‐experienced group” included surgeries from the 1st to the 10th case, while the “experienced group” included surgeries from the 11th case and beyond. All statistical analyses were calculated using Stata/IC 16 for Mac (StataCorp, Texas, USA), with a *p*‐value of <0.05 indicating statistical significance. We calculated between‐group differences using Mann–Whitney *U* test for continuous variables.

## 3 RESULTS

### 3.1 Step 1: The establishment of automatic instruments recognition using AI

We annotated 540 Cadiere forceps, 898 Fenestrated bipolars, 359 Suction tubes, 307 Maryland bipolars, 688 Harmonic scalpels, 400 Staplers, and 59 Large clips in both the training and test data, respectively. Table 1 shows the comparison of IoU and accuracy for each instrument. The average IoU was 0.82 ± 0.12 and ranged from 0.56 to 0.92. The IoU was the highest in the Maryland bipolar, following the Large clip and Suction tube. The average accuracy was 87.1 ± 11.9% and ranged from 61.2% to 95.3%. The suction tube had the highest accuracy, followed by Harmonic scalpel and Maryland bipolar which also showed high accuracy. For the purposes of machine learning, it is a common consensus and industry standard to consider a value of greater than 0.5 as a good IoU and greater than 70% as a good accuracy score.^34^ For this study, almost all instances of our results were above these thresholds.

**TABLE 1:** The comparison of IoU and accuracy for each device.

Device	IoU	Accuracy (%)
Cadiere forceps	0.56	61.2
Fenestrated bipolar	0.83	88.7
Suction tube	0.88	95.3
Maryland bipolar	0.92	93.2
Harmonic scalpel	0.86	93.8
Stapler	0.81	85.9
Large clip	0.90	91.6

	Average IoU	Average accuracy (%)
	0.82 ± 0.12	87.1 ± 11.9

**FIGURE 1:**
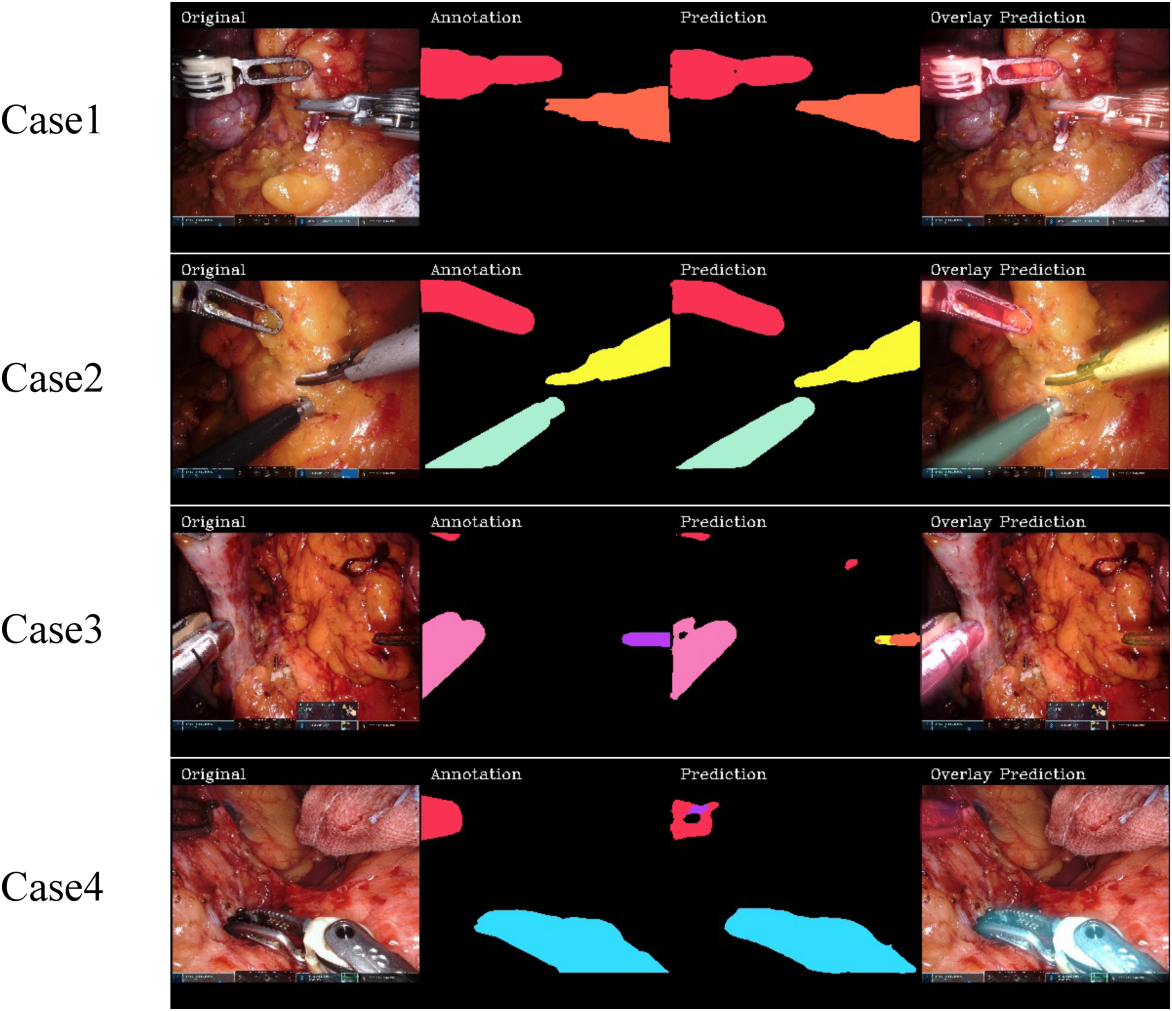
Overlay prediction using AI for four representative cases.

To visualize the predictive accuracy of our model, Figure 1 showed overlay prediction for four representative cases. The first two cases achieved nearly complete agreement between ground truth data and predicted segmentation. On the other hand, the latter two cases misidentified instruments that were slightly visualized in the images as different types of instruments.

### 3.2 Step 2: The comparison between experienced and non‐experienced surgeons based on usage of instruments during infrapyloric LND

#### 3.2.1 Patient characteristics

The comparison of characteristics between experienced and non‐experienced groups were presented in Table 2. Although the experienced group performed more D2 lymphadenectomy cases (*p* = 0.011), operative time was significantly shorter (344 ± 80 min in the non‐experienced group, 260 ± 87 min in the experienced group), and total complications were less in the experienced group (*p* = 0.008). Although complications such as pancreatic fistula and postoperative bleeding tended to decrease among the experienced surgeons, each complication was not significantly different between the two groups. The other factors such as sex, age, BMI, tumor location, tumor circumference, and intraoperative blood loss were not significantly different between experienced and non‐experienced surgeons.

**TABLE 2:** The comparison of characteristics between experienced and non‐experienced group.

Factor	Surgeons		*p* Value
	Non‐experienced group (*n* = 22)	Experienced group (*n* = 33)	
Sex (male/female)	18/4	26/7	0.783
Age	65.2 ± 11.4	68.9 ± 10.8	0.216
Body mass index (kg/m^2^)	22.8 ± 3.1	22.3 ± 3.1	0.481
Tumor location (U/M/L)	2/8/12	3/17/13	0.513
Tumor circumference (Ant/Post/Gre/Less/Total)	4/6/6/6/0	8/7/9/8/1	0.888
cStage (I/II/III)	21/1/0	25/7/1	0.150
Lymphadenectomy (D1 + /D2)	7/15	2/31	0.011
Operative time (min)	344 ± 80	260 ± 87	0.001
Intraoperative blood loss (mL)	24 ± 41	18 ± 30	0.479
Postoperative complications	6	1	0.008
Postoperative bleeding	2	0	0.078
Pancreatic fistula	1	0	0.216
Anastomotic leakage	1	0	0.216
Dumping syndrome	1	0	0.216
Atelectasis	0	1	0.410
Pulmonary embolism	1	0	0.216

#### 3.2.2 Instruments usage in infrapyloric lymphadenectomy

**FIGURE 2:**
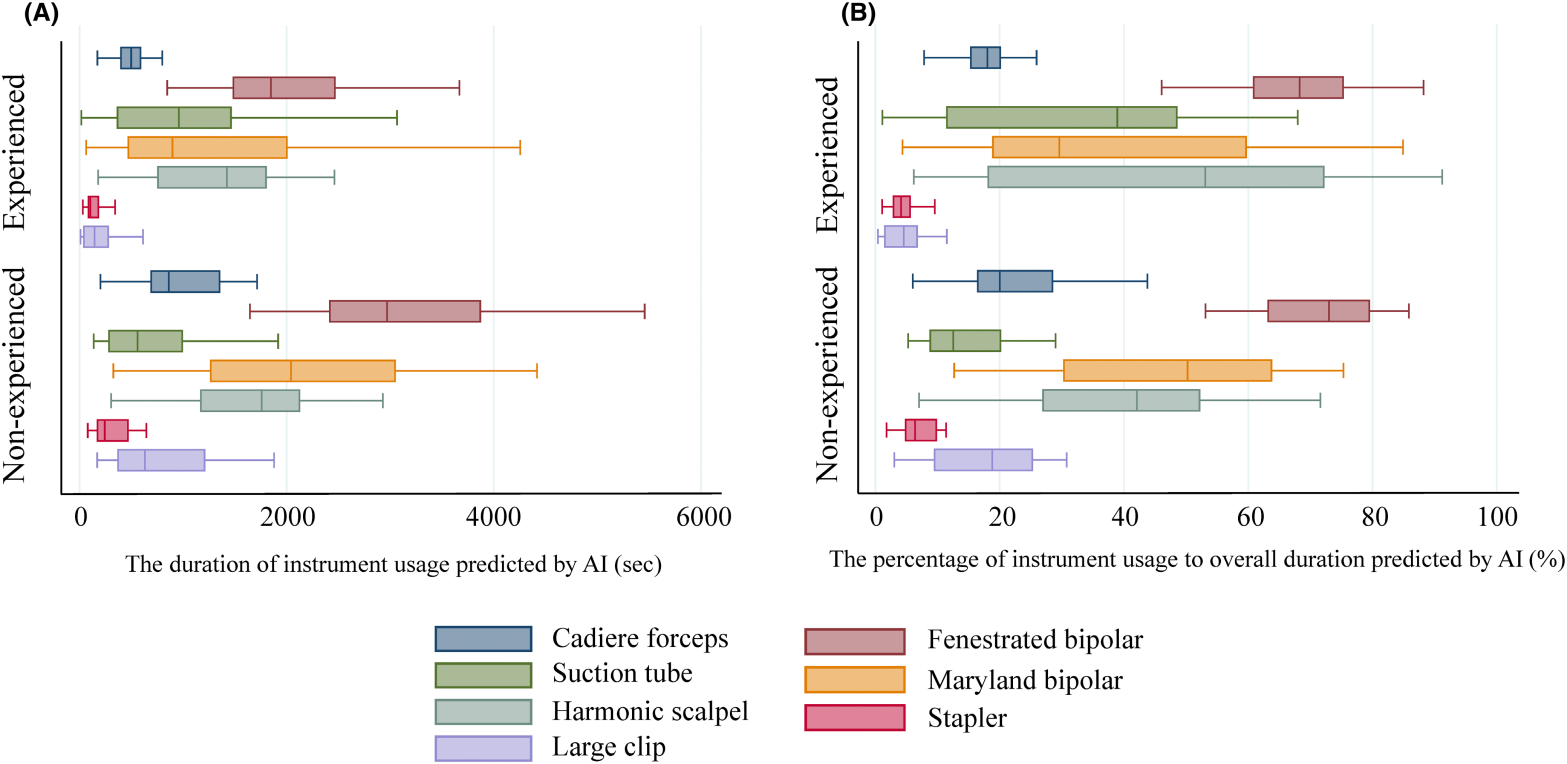
The box plots for instruments usage in infrapyloric lymphadenectomy.

A comparison of the duration of instrument usage predicted by AI during infrapyloric lymphadenectomy between experienced and non‐experienced surgeons are shown in Figure 2 and Table 3. All instruments except the Suction tube and Harmonic scalpel were detected to be used significantly longer in non‐experienced compared to experienced group.

**TABLE 3:** The comparison for duration of instrument usage predicted by AI between experienced and non‐experienced group during infrapyloric lymphadenectomy.

Device	The duration of instrument usage predicted by AI (sec)		*p* Value	The percentage of instrument usage to overall duration predicted by AI (%)^a^		*p* Value
	Non‐experienced group (*n* = 22)	Experienced group (*n* = 33)		Non‐experienced group (*n* = 22)	Experienced group (*n* = 33)	
Cadiere forceps	994 ± 565	599 ± 338	0.002	22.2 ± 9.3	17.9 ± 4.8	0.083
Fenestrated bipolar	3112 ± 1011	2242 ± 1166	<0.001	71.0 ± 9.4	67.0 ± 10.2	0.223
Suction tube	749 ± 634	1050 ± 820	0.186	15.6 ± 11.3	33.0 ± 20.9	0.010
Maryland bipolar	2183 ± 1138	1454 ± 1435	<0.001	48.4 ± 19.6	37.6 ± 24.6	0.059
Harmonic scalpel	1793 ± 928	1332 ± 638	0.071	41.3 ± 18.0	47.6 ± 27.2	0.303
Stapler	343 ± 282	161 ± 127	<0.001	8.0 ± 6.1	4.7 ± 2.5	0.011
Large clip	797 ± 544	198 ± 175	<0.001	17.6 ± 9.2	5.7 ± 4.9	<0.001

^a^ The percentage of each instrument usage to overall infrapyloric lymphadenectomy duration predicted by AI.

Table 3 also shows the results of the comparison for the percentage of each instrument's usage to overall infrapyloric lymphadenectomy duration which is predicted by AI. The use of Stapler and Large clip were significantly shorter in the experienced compared to the non‐experienced group. Nevertheless, the usage of the Suction tube, even though it was not significantly different, was longer in the experienced group than in the non‐experienced group.

## 4 DISCUSSION

In the present study, we demonstrated that AI can both be used to successfully identify and outline the surgical instruments used in RDG with accuracy using novel instance segmentation. We further concluded that our AI model can accurately predict the surgical skill of a surgeon performing RDG by analyzing this surgical instrument usage. This technology allows for an unbiased, reproducible, automated assessment of the procedure that can be used to both evaluate and train physicians to provide the highest quality operation for patients.

Other studies have similarly demonstrated that instrument tool usage is correlated with surgical skill. One study focused on surgical skill in laparoscopic gastrectomy using AI and found that usage time for the dissecting forceps and hemostatic clip appliers was greater for unqualified surgeons when compared to surgical experts. Furthermore, energy device instruments also demonstrated higher intraoperative use time for non‐expert surgeons.^28^ These results were consistent with our findings in RDG cases, as energy devices such as Stapler and Large clip instrument time usage increased significantly for non‐experienced when compared to experienced group, while the usage time of suction tube decreased for non‐experienced surgeons. However, our approach adds additional findings compared to the previous study. Primarily, this study was conducted with robotic surgery because the technique can be evaluated without reflecting the assistant's technique or assistant's instrument movements. Moreover, we not only chosed to study the rapidly growing *robotic* distal gastrectomy procedure instead of *laparoscopic* gastrectomy, but we also implemented a far more precise method of recognition. Yamazaki et al. used bounding boxes, or rectangular fields, to identify the surgical tools while our AI system uses object‐specific recognition and annotation to generate outlines around the surgical tools. This technology has potential for future studies using instrument position to analyze surgical skill. The precise delineation of details such as the tip of instruments or blades could potentially contribute to avoiding organ damage during surgery in the future, making segmentation more valuable than using bounding boxes. Furthermore, unlike detection using bounding boxes as in previous studies, applying instance segmentation can reduce the number of images required for building an AI model.^28^

In the current study, the duration of almost all instruments usage predicted by AI was longer in the non‐experienced group. The duration of instrument usage alone does not fully reflect the surgical skill. However, we think that it may be more useful for evaluating skill than total operative time including redundant procedures such as instrument docking and camera cleaning which are not related to surgical skill. Therefore, we focused on duration of instruments which show one aspect of surgical skill that we can evaluate precisely. Nevertheless, the duration of instrument usage is strongly correlated with total operative time, so we focused on the ratio of each instrument usage.

In this study, experienced surgeons used the Large clip and the Stapler more frequently. This is because clipping vessels with the Large clip and duodenal resection with the Stapler are highly demanding steps in the procedure which reflects surgical experience. Though the percentage of the Suction tube usage was higher in the experienced group due to the fact that the instrument was operated by assistant surgeons, we do not believe it directly relates to surgical skill. Although there was no significant difference, Cadiere forceps also tended to be used less frequently in the experienced group. This may be because experienced surgeons can create the field of view more efficiently and therefore require fewer field changes. Maryland bipolar may be preferred by non‐experienced surgeons because of its large tip range of motion and relatively ease of control. There was no significant difference in the percentage of Harmonic scalpel and Fenestrated bipolar usage, suggesting that the use of these instruments is not related to surgical skill. The analysis of instrument usage pattern allows us to evaluate the surgical learning curve simply and precisely while providing us with a metric to measure surgical skill. Moreover, focusing on instrument usage patterns in both experienced and non‐experienced surgeon groups may also lead to improved surgical skills in non‐experienced surgeons by understanding how more experienced surgeons perform operations.

These findings have significant potential to affect the field of RDG training and education. For example, an AI surgical skill assessment could be used to review each RDG performed by a non‐experienced surgeon, and this information could be used as a tool to accelerate the surgical learning curve associated with this complex procedure. This technology also has the potential to automate and enhance education for trainees along with providing continuous evaluation and quality‐control of this operation for different surgeons.

Limitations of this study include that this is a retrospective trial using recorded RDG from a single institution. Also, only the da Vinci robotic systems were used and other systems such as the Hinotori Surgical Robot were not studied here. The accuracy of our study could further be increased with a larger set of training data since the training data size is directly correlated with segmentation mask IoU scores and instrument recognition accuracy scores. It should be noted that our IoU and accuracy results are impressive given the relatively small training data set since other models have needed more than three times the number of images.

In conclusion, we demonstrated that AI can be used to successfully identify and outline the surgical instruments used in RDG with accuracy using novel instance segmentation. We further concluded that our AI model can accurately predict the surgical skill of a surgeon performing RDG by analyzing surgical instrument usage. It is important to evaluate the surgical quality of each step as a component of the overall surgical skill along with analyzing each step's duration. So far, several reports suggested that specific surgical phases and tool movement are significantly associated with surgical skill and learning curve.^23^, ^25^, ^26^, ^27^, ^31^, ^35^ Therefore, we believe that not only the duration of instrument usage but also other factors such as surgical phase, instrument movement, and preoperative clinical information will result in establishing future AI models with high accuracy. Finally, it is important to note that surgical speed may be mistaken for surgical efficiency and skill, so future AI models may need to incorporate patient outcomes and complications in order to enhance the predictive capabilities of this tool to both evaluate and train surgeons.

## AUTHOR CONTRIBUTIONS

Literature search: JS, TF, MT, HK. Study design: JS, TF, MT, HK, YM, SM, KF, RN, YK. Analysis plan: JS, TF, MT, HK, YK. Data analysis and interpretation: JS, TF, MT, HK, YM, SM, KF, RN, YK. Drafting of manuscript: JS, TF, MT, HK. Manuscript revision: YK.

## FUNDING INFORMATION

The present study was not funded by any organization.

## CONFLICT OF INTEREST STATEMENT

James S. Strong, Tasuku Furube, Hirofumi Kawakubo, Yusuke Maeda, Satoru Matsuda, Kazumasa Fukuda, and Rieko Nakamura have no conflicts of interest or financial ties to disclose. Yuko Kitagawa received lecture fees from CHUGAI PHARMACEUTICAL CO., LTD., TAIHO PHARMACEUTICAL CO., LTD, ASAHI KASEI PHARMA CORPORATION, Otsuka Pharmaceutical Factory Inc., SHIONOGI & CO., LTD., Nippon Covidien Inc., ONO PHARMACEUTICAL CO., LTD., Bristol‐Myers Squibb K.K. Author Y.K was supported by grants from CHUGAI PHARMACEUTICAL CO., LTD., TAIHO PHARMACEUTICAL CO., LTD, Yakult Honsha Co. Ltd., AsahiKASEI Co., Ltd., Otsuka Pharmaceutical Co., Ltd., Takeda Pharmaceutical Co., Ltd., ONO PHARMACEUTICAL CO., LTD., TSUMURA & CO., Kyouwa Hakkou Kirin Co., Ltd., DAINIPPON SUMITOMO PHARMA Co., Ltd., EA Pharma Co., Ltd., Astellas Pharma Inc., Toyama Chemical Co., Ltd., MEDICON INC., KAKEN PHARMACEUTICAL CO. LTD., Eisai Co., Ltd., Otsuka Pharmaceutical Factory Inc., TEIJIN PHARMA LIMITED., NIHON PHARMACEUTICAL CO., LTD., and Nippon Covidien Inc. Author Y.K held an endowed chair provided by CHUGAI PHARMACEUTICAL CO., LTD. and TAIHO PHARMACEUTICAL CO., LTD, outside the submitted work. Masashi Takeuchi has stocks from Direava inc. outside the submitted work. Author Y.K is a current chief editor of *Annals of Gastroenterological Surgery* while none of the other authors of this article is a current Editor or Editorial Board Members of *Annals of Gastroenterological Surgery*. All authors' meets the authorship criteria and all authors are in agreement with the content of the article.

## ETHICS STATEMENT

Approval of the research protocol: This study was conducted with the approval of the Ethics Committee of Keio University School of Medicine (Approval Number: 20210034).

Informed Consent: The opt‐out method to obtain patient consent was utilized.

Registry and the Registration No. of the study/trial: N/A.

Animal Studies: N/A.
